# Effects of population structure on genetic association studies

**DOI:** 10.1186/1471-2156-6-S1-S109

**Published:** 2005-12-30

**Authors:** Hongyan Xu, Sanjay Shete

**Affiliations:** 1 Department of Epidemiology, Unit 1340, University of Texas M. D. Anderson Cancer Center, 1155 Hermann Pressler Boulevard, Houston, TX 77030, USA

## Abstract

Population-based case-control association is a promising approach for unravelling the genetic basis of complex diseases. One potential problem of this approach is the presence of population structure in the samples. Using the Collaborative Study on the Genetics of Alcoholism (COGA) single-nucleotide polymorphism (SNP) datasets, we addressed three questions: How can the degree of population structure be quantified, and how does the population structure affect association studies? How accurate and efficient is the genomic control method in correcting for population structure? The amount of population structure in the COGA SNP data was found to inflate the *p*-value in association tests. Genomic control was found to be effective only when the appropriate number of markers was used in the control group in order to correctly calibrate the test. The approach presented in this paper could be used to select the appropriate number of markers for use in the genomic control method of correcting population structure.

## Background

Unraveling the genetic basis of psychiatric diseases such as alcoholism is becoming the major challenge and focus of genetic studies, and large-scale case-control association studies at the genomic level are a promising approach. One potential problem for association studies is the presence of population structure in the samples, which raises the potential for confounding and spurious results. For example, if the samples come from several subpopulations with different allele frequencies, and if the proportions of cases and controls sampled from each subpopulation are not matched, differences in allele frequencies between cases and controls will appear, mimicking a statistical signal of association and leading to false-positive results. However, there has been much debate over how much population structure exists and how serious a problem it poses to association studies. With the advances in genotyping techniques, association studies can now be carried out at the genomic level using thousands of genetic markers. There have been few studies of the effects of population structure on association studies using such data. Recently, Marchini et al. performed such a study [1]; however, their results were based on simulated samples using a Bayesian model extrapolated from a very limited dataset. Even though the Bayesian model fit their data quite well, it would be of interest to compare their results with those from a study that uses a large set of real data. Therefore, we used the Collaborative Study on the Genetics of Alcoholism (COGA) data from Genetic Analysis Workshop 14 (GAW14) to assess the effects of population structure on large-scale association studies. Three questions were addressed by our study. How can the degree of population structure be quantified, and how does the population structure affect association studies? How accurate and efficient is the genomic control method for correcting for population structure?

## Methods

### Data

The COGA single-nucleotide polymorphism (SNP) data from GAW14 was used in this study. The data consisted of two sets of SNP genotype data, one from Affymetrix, the other from Illumina. The datasets contained individuals from 143 extended families. A total of 304 unrelated individuals were selected from these families, including the founders and marry-ins from each family. When the genotypes of both founders were not available, one of the children of the founders was randomly selected. Among these 304 unrelated individuals, 265 were White, 30 were Black, and 9 were others. The Affymetrix dataset contained genotypes at 10,810 SNP markers, while the Illumina dataset contained genotypes at 4,596 markers.

### Quantifying population structure

To quantify the population structure, we used the statistic *F*_*ST*_, which measures variation in allele frequency between populations. We used the unbiased estimator of *F*_*ST *_at a bi-allelic SNP described by Weir and Cockerham [2] (see also Weir [3]). Specifically, suppose samples are drawn from *S *populations and there are two alleles, A and a, at any given SNP. Let the frequency of allele A in the *i*^th ^population be *p*_*i*_, the average allele frequency across populations be , and the sample size from the *i*^th ^population be *n*_*i*_. Then the observed mean square errors of allele frequency within a population, denoted as MSI, was computed as



and the observed mean square errors of allele frequency between populations, denoted as MSP, was computed as,



*F*_*ST *_can then be estimated as,



where *n*_*c *_is the average sample size across populations. To correct for the different sample sizes from each population, and is given by



It is possible for this unbiased estimator to result in values below zero; therefore, because *F*_*ST *_must be a value between 0 and 1, *F*_*ST *_is set to 0 in such situations. We estimated values of *F*_*ST *_for each SNP in our sample of 304 individuals.

### Measuring association at an SNP locus

In order to quantify the effects of population structure on tests for association in our sample, we randomly assigned case/control status while keeping the population structure observed in the total sample. We randomly chose 152 Whites to be cases, and the remaining individuals were assigned to be controls. For each SNP locus, 1,000 such random assignments were performed. The association for each assignment was measured using Armitage's trend test under an additive genetic model [4], which has been shown to be robust against deviations of genotype frequencies from Hardy-Weinberg equilibrium [5]. Suppose the two alleles at an SNP locus are denoted as A and a, then this test statistic is given by



where *N *is the total sample size, *R *is the number of cases, *n*_1 _and *n*_2 _are the number of individuals with genotypes Aa and AA in the sample, respectively, and *r*_1 _and *r*_2 _are the number of cases with genotypes Aa and AA, respectively. Under the null hypothesis of no association, and assuming no population structure, *Y*^2 ^should follow asymptotically a *χ*^2 ^distribution with 1 degree of freedom. For each locus, 1,000 random assignments were performed, and *Y*^2 ^was computed for each assignment. Thus, using the Affymetric data set we generated 10,810,000 samples from the distribution of *Y*^2 ^with the specific level of population structure observed in the COGA data. For the Illumina data set, we generated 4,596,000 such samples. The empirical distribution of the test statistic *Y*^2 ^was compared with the  distribution to study the effects of population structure under the null hypothesis.

### Genomic control

Recently, several statistical methods have been proposed to perform association studies in the presence of population structure. One popular method is genomic control, in which a set of unlinked markers is selected to correct for population structure [6]. The idea is that population structure inflates the test statistic *Y*^2 ^by some constant value, *λ*, which can be estimated by the *L *unlinked markers in the genomic control group. Here we examined the performance of genomic control with a large sample size and small nominal *p*-value from the real COGA dataset. The *L *markers were chosen randomly from all the markers in each dataset by assuming a uniform distribution over the markers, with the constraint that the genetic distance between neighboring markers was greater than 1 cM. This selection strategy leads to loci in genomic control that are unlikely to be correlated [7]. As in the study by Bacanu et al. [8], a robust estimator of *λ *was used, which is given by . As in another recent study [1], any estimate of *λ *below 1 was set to 1.

## Results

The mean of *F*_*ST *_in the Affymetrix data set was 0.085 with a variance of 0.013. The mean of *F*_*ST *_in the Illumina data set was 0.070 with a variance of 0.006. These results indicate that there is a substantial amount of population structure in our samples. The results are similar to values reported in major human races [9].

**Figure 1:**
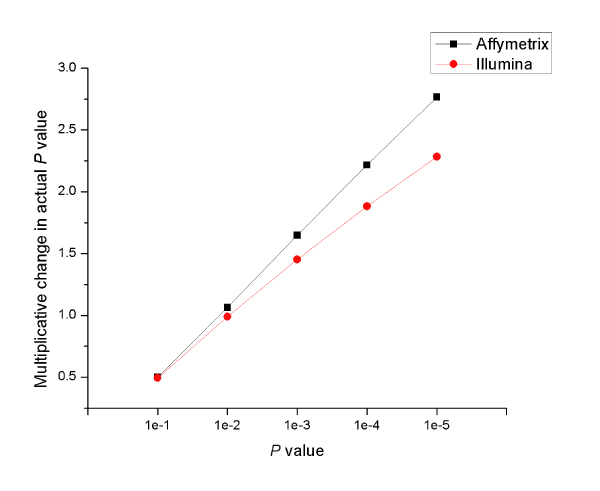
Multiplicative changes in *p*-values due to population structure.

The effects of population structure on association studies were assessed by comparing the actual *p*-value from the empirical distribution of *Y*^2 ^with its nominal *p*-value from the  distribution. The multiplicative change of the resulting *p*-value is defined as the actual *p*-value divided by the nominal *p*-value. The results from the Affymetrix and Illumina datasets are given in Figure 1, in which the multiplicative change in *p*-values is graphed on a log_10 _scale. Figure 1 indicates that the level of population structure in our sample would cause problems for conventional association studies. The actual *p*-value was inflated owing to population structure. This suggests that the presence of population structure produces an empirical distribution of *p*-values with heavier tails than the theoretical distribution, which leads to more false-positive results. Also, the problem posed by population structure becomes more and more serious as the nominal *p*-value decreases. When nominal *p *= 10^-5^, the actual *p*-value was inflated by more than 2 orders of magnitude as it is shown on a log_10 _scale. The magnitude of this inflation was different in the 2 datasets, which could be due to the differences in the number of markers and allele frequencies of the markers.

**Figure 2:**
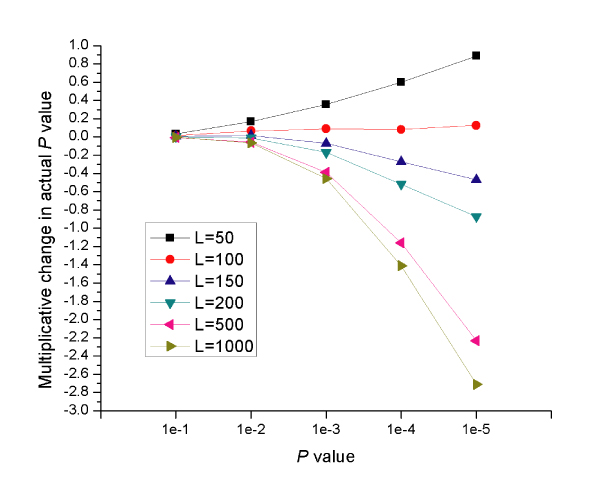
Multiplicative changes in *p*-values with genomic control in Affymetrix dataset.

Genomic control was performed with various numbers of markers, *L*, examined in the genomic control group. Results from the Affymetrix dataset are shown in Figure 2. The results from the Illumina dataset were quite similar, and are not shown. These results indicate that genomic control can be an effective approach to correct for population structure. Compared to the corresponding values in Figure 1, the actual *p*-value in Figure 2 decreased substantially. However, the effectiveness of genomic control varies depending on *L*, the number of markers examined. When *L *is small (e.g., *L *= 50), the actual *p*-value will still be inflated compared to its nominal *p*-value, most obviously when the nominal *p*-value is very small. When *L *is large (e.g., *L *= 500 or 1,000), the effects of population structure can be over-corrected, especially when the nominal *p*-value is small. For example, when *L *= 1,000, the actual *p*-value after correction can be more than 2 orders of magnitude smaller than the nominal *p*-value when *p *= 10^-5^, which makes the test unacceptably conservative, leading to the loss of power to detect a true signal. Therefore, it is not always true that the more loci in the genomic control group, the better its correction. The number of loci *L *used for genomic controls has to be carefully selected. In our case, *L *= 100 consistently resulted in good correction at various levels of *p*-values. It took an average of 2 hours to perform the marker selection and genomic control for each *L *on our Sun Sparc system with a 750 MHz CPU.

## Discussion

Figure 1 indicates that the amount of population structure in our sample of unrelated individuals drawn from the COGA families could well inflate the *p*-values of genetic association studies. This result is the opposite of that obtained by Marchini et al. [1], as illustrated in their Figure 3, where population structure was shown to decrease *p*-values. The discrepancy could be due to the small sample sizes used in their study.

The results in Figure 1 also indicate that as the nominal *p*-value decreases, the problem posed by a given amount of population structure becomes more and more serious. This could have important implications for genetic studies in which very large numbers of markers are used. Owing to the thousands of markers tested in such studies, correcting for multiple testing would mean that any "significant" result would have a much lower *p*-value in order for the association results to be considered "significant." Usually, the genome-wide significance level is set in the very low range of 10^-4 ^to 10^-8 ^[10]. Our results indicate that in this range, the problem posed by population structure becomes very serious indeed. As a consequence, the effects of population structure cannot be safely ignored for genome-wide association studies, and steps must be taken to correct for the effects of population structure.

One popular method for correcting the effects of population structure is the genomic control approach, wherein several unlinked markers are genotyped to correct for the observed level of population structure in the sample at hand. The performance of genomic control was assessed in our samples for a variable number of independent markers. The performance of genomic control does vary, depending on the number of markers *L *examined in the genomic control group. When *L *is small (e.g., *L *= 50), the correction is incomplete, resulting in a lax test and false-positive results. When *L *is large, there is over-correction, resulting in a conservative test, which would lead to missing real signals. The conclusion by Marchini et al. [1] that "If enough loci are used, then the test will typically be approximately calibrated" does not seem to be true according to our analysis. Therefore, choosing the appropriate *L *becomes critical for correctly calibrating tests for association. The exact reason for this result is unclear, and further validation is required. Simulation studies have suggested that linkage disequilibrium is not likely to extend beyond 5 kb, even in relatively isolated populations [7]. Since in our study, the genetic distance between neighboring markers was at least 1 cM, using the approximation 1 cM = 1 Mb, it is unlikely that correlation between markers could be the reason. Therefore, one possible explanation is that in our dataset, there could be much variation in *λ *across the genome, which is not accounted for in the estimation of *λ*. Following the procedure proposed in this report, a grid search could be performed for the appropriate *L *at a specific level of significance.

## Conclusion

Through our analysis based on real datasets, we have shown that population structure inflates the *p*-values in genetic association studies, especially in cases of very small *p*-values. Therefore, the effects of population structure cannot be safely ignored in large-scale association studies at the genomic level, where the *p*-value is usually required to be very small in order to achieve statistical significance. Genomic control is an effective way to correct for the effects of population structure, but only when the appropriate number of markers is used. The approach proposed in this paper could be used to select the appropriate number of markers. However, caution must be taken because the exact underlying reason for varying the number of loci in genomic controls may be dependant on several other factors that were not considered here.

## Abbreviations

COGA: Collaborative Study of the Genetics on Alcoholism

GAW14: Genetic Analysis Workshop 14

MSI: Mean square errors of allele frequency within a population

MSP: Mean square errors of allele frequency between a population

SNP: Single-nucleotide polymorphism

## Authors' contributions

HX conceived of the study, participated in the design of the study, and performed the statistical analysis. SS participated in its design and coordination and helped to draft the manuscript. All authors read and approved the final manuscript.
